# West Nile Virus in Horses, sub-Saharan Africa

**DOI:** 10.3201/eid1212.060042

**Published:** 2006-12

**Authors:** Olivier Cabre, Marc Grandadam, Jean-Lou Marié, Patrick Gravier, Aurélie Prangé, Yan Santinelli, Vincent Rous, Olivier Bourry, Jean-Paul Durand, Hugues Tolou, Bernard Davoust

**Affiliations:** * École du Val-de-Grâce, Paris, France;; † Institut de Médecine Tropicale du Service de Santé des Armées, Marseille, France;; ‡ Secteur Vétérinaire de Marseille, Marseille, France;; § Service Vétérinaire du Régiment de Cavalerie de la Garde Républicaine, Paris, France;; ¶ Secteur Vétérinaire de Lyon, Lyon, France;; # Centre International de Recherches Médicales, Franceville, Gabon;; ** Direction Régionale du Service de Santé des Armées de Toulon, Toulon, France

**Keywords:** West Nile virus, Horses, Africa, Senegal, Côte d’Ivoire, Chad, Democratic Republic of the Congo, Gabon, Djibouti, Western blot, dispatch

## Abstract

To evaluate the presence and extension of West Nile virus where French soldiers are stationed in Africa, specific antibody prevalence was determined by using ELISA and Western blot. Among 245 horses living in close proximity to the soldiers, seroprevalence was particularly high in Chad (97%) and Senegal (92%).

West Nile virus (WNV), a mosquito-transmitted flavivirus, was first isolated in Africa, West Nile district of Uganda, in 1937 (*1*). It has been shown to infect humans and a wide spectrum of animal species, including birds and horses. WNV infection is often inapparent or mild in humans but may cause severe and even fatal encephalitis in horses (*2*). Since 1999, dissemination of the virus through North America has reinforced interest in WNV epidemiology and evolution. Before 1999, outbreaks have been reported in North Africa, Israel, Romania, Russia, and France, where the virus may have been imported by migratory birds (*3**–**5*). However, few data are available on the current circulation of WNV in sub-Saharan Africa because of lack of surveillance and diagnostic tools in those countries.

Assessing and preventing human and zoonotic infectious diseases in tropical areas, particularly Africa, are essential missions of the French Defense Medical Service. To evaluate the presence and extension of WNV in the sub-Saharan African areas where French soldiers are stationed, serologic surveillance of horses living in close proximity was initiated in 2002.

## The Study

**Figure 1:**
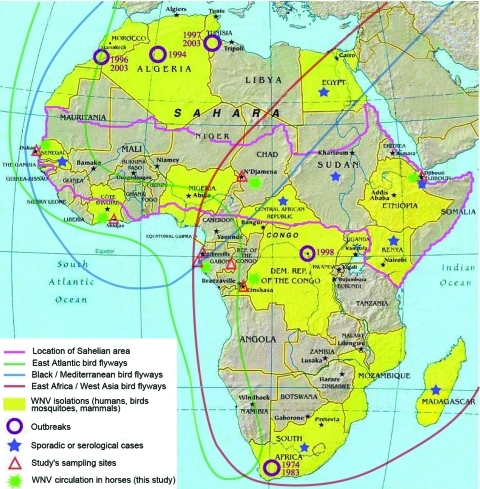
West Nile virus (WNV) circulation in Africa (*3**,**6**–**10*). Map of Africa summarizes published data related to WNV isolations, outbreaks, and sporadic or serologic cases (including this study). It also indicates the main bird migration routes (source: Wetlands International, Wageningen, the Netherlands). Source: Food and Agricultural Organization of the United Nations.

From December 2002 through August 2005, blood samples were collected from 245 horses in 13 riding stables located in Senegal (Dakar, n = 25), Côte d'Ivoire (Abidjan, n = 95), Chad (N'Djamena, n = 30), Democratic Republic of the Congo (Kinshasa, n = 20), Gabon (Libreville, Port Gentil, and Moanda, n = 64), and Djibouti (Djibouti, n = 11) (Figure 1). Some horses were sampled twice in Chad (n = 18) and in Côte d'Ivoire (n = 18) during a period of 11–13 months. Origin, travel history, and how long the tested horses lived in the studied areas were not well known, but the horses were generally born and bred in the countries from which they were sampled (some of them in neighboring countries such as Burkina Faso, Mali, Niger, and Ethiopia), and none had a history of WNV vaccination.

**Figure 2:**
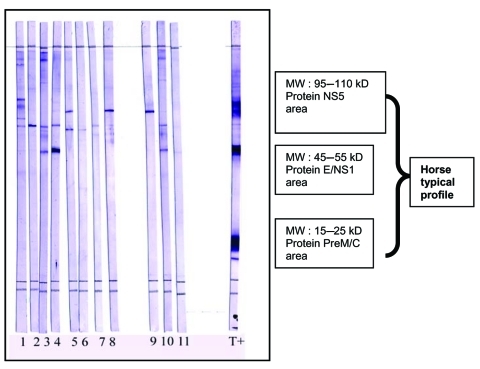
West Nile virus (WNV) Western blot as a validation of ELISA-positive results. Proteins from WNV-infected cells lysates were separated by sodium dodecyl sulfate–polyacrylamide gel electrophoresis on 13% acrylamide gels and transferred on polyvinylidene difluoride membranes. Strips were cut and blocked with nonfat milk (5%). Serum samples diluted (1:200) in phosphate-buffered saline (PBS), 5% nonfat milk, and 0.1% Tween 20 were loaded on strips and incubated for 1 h with slow shaking. Strips were washed 4 times in PBS, 0.5% Tween, and incubated for 1 h in anti-horse immunoglobulin G (IgG) peroxidase (1:8,000). After 4 washes, Western blots were incubated for 5 min with trimethylbenzidine substrate (with specific enhancer). Strips were washed once with water to stop the staining. Results were obtained within 24 h. MW, molecular weight; T+, horse from Chad with positive Western blot results; serum samples 1–11, horse from southeast France, IgG positive by ELISA but negative by neutralization; NS5, nonstructural protein 5; E, envelope; PreM, premembrane; C, capsid.

Blood was centrifuged within 24 hours after collection. Serum was separated, frozen at –20°C, and sent to the virology laboratory of the Institut de Médecine Tropicale du Service de Santé des Armées in Marseille, France. Each sample was systematically tested for WNV-specific immunoglobulin G (IgG) by using an ELISA made in house. Antigen was prepared from a crude supernatant of Vero cells collected after 4 days of infection with WNV reference strain Eg 101 (viral titer >10^7^ ID/mL) and treated with 1% Triton 100 and β-propiolactone (1:1,000). IgG was detected by using commercial peroxidase anti-horse IgG and tetramethylbenzidine as the substrate and standard procedures of ELISA capture. Serum specimens were considered positive for IgG when the optical density (OD) in antigen-positive wells was >0.3 and the ratio between the OD in corresponding antigen-positive wells and the mean OD in antigen-negative wells was >3.5. Because of the antigenic cross-reactivity among viruses of the *Flavivirus* genus, validation of ELISA IgG–positive samples was necessary. The plaque reduction neutralization test (PRNT) is the serologic reference method. All the IgG-positive sera collected during 2002–2003 were tested as described (*11*). In a 96-well plate, 4 dilutions of each serum sample (1:10, 1:40, 1:160, 1:640; 4 wells for each dilution) were incubated at 37°C for 1 hour in a viral suspension of 10–50 PFU in 50 μL before the addition of 100 μL of a Vero cell suspension (4×10^4^/well). Four days later, the cell layer was fixed in formol and stained with crystal violet. A test result was considered positive for a dilution if the plaque reduction was >90% compared with the negative control. Because this method is fastidious and slow, we have used an alternative Western blot (WB) approach as described in Figure 2 (*12*).

Complete (100%) correlation between WB and PRNT and high specificity of WB were observed for a panel of 79 serum samples. Thus, only WB was used for validation of ELISA IgG-positive sera for the 2004–2005 samples. All serum samples that were positive for WNV IgG were further investigated using immunocapture IgM ELISA to evaluate the time of infection.

## Conclusions

Except in Gabon (3%), high seroprevalence (28%–97%) for WNV was detected in horses in West Africa and Central Africa, especially in N'Djamena (97%) and Dakar (92%) (Table 1). Seroprevalence of 9% was detected in East Africa (Djibouti).

**Table 1:** West Nile virus antibody prevalence in horses in 6 African countries, December 2002–August 2005*

Country (sampling sites)	Sampling date (no. riding stables)	No. tested	No. IgG+†	No. confirmed IgG+‡	% Seroprevalence
Senegal (Dakar)	Dec 2002, Apr 2003 (1)	25	23	23	92
Côte d'Ivoire (Abidjan)	Dec 2003, Dec 2004, Jan 2005 (3)	95	51	27	28
Chad (N'Djamena)	Nov 2003, Oct 2004 (2)	30	29	29	97
DRC (Kinshasa)	Jul 2004 (1)	20	9	6	30
Gabon (Libreville, Port Gentil, Moanda)	Dec 2004 (4)	64	9	2	3
Djibouti (Djibouti)	Jul 2004, Aug 2005 (2)	11	2	1	9
Total		245	123	88	36

*IgG+, positive for immunoglobulin G; DRC, Democratic Republic of the Congo. †By ELISA. ‡By Western blot and seroneutralization for samples from 2002–2003, by Western blot only for samples from 2004 to 2005.

All horses positive for IgG were negative for IgM, which indicates relatively old infection. Estimating the date of onset of WNV infection is difficult because of a lack of published data relative to WNV IgM and IgG response in naturally infected horses; only persistence of IgG several years after infection has been described (*4*). Because histories of tested horses are not well known, determining precisely when and where horses became infected is difficult. However, infections likely occurred in sampling countries or neighboring sub-Saharan African countries.

Seroconversion from negative to positive was found in 2 horses in Chad (Table 2) from 2003 through 2004, while 5 of 15 seropositive horses became seronegative, which suggests maintenance of an enzootic cycle in this area but at a low level. During the same period in Côte d'Ivoire, 9 of 10 previously seropositive horses were seronegative, while none of seronegative horses became seropositive. The most probable explanation is a decrease in IgG titer under the retained threshold of positivity compatible with the decrease of WNV IgG response in horses, which suggests the presence of an older epizootic in this area.

**Table 2:** Results of follow-up testing for West Nile virus in horses in Chad and Côte d'Ivoire

	Initial testing*	Follow-up testing†
Chad (n = 18)	3 negative	1 negative, 2 positive
	15 positive	5 negative, 10 positive
Côte d'Ivoire (n = 18)	8 negative	8 negative
	10 positive	9 negative, 1 positivE

*November 2003 in Chad and December 2003 in Côte d'Ivoire. Positive results were confirmed by Western blot and seroneutralization. †October 2004 in Chad and December 2004–January 2005 in Côte d'Ivoire. Positive results were confirmed by Western blot.

The immunoblotting method is a fast and specific confirmation assay for validation of ELISA WNV IgG–positive sera. Once validated by further studies, WB could be used as an alternative to PRNT.

Serologic data from our study should be considered as evidence of WNV activity in sub-Saharan Africa, which has a potential risk for populations and foreigners, including French soldiers. Previously, WNV was known to circulate in mosquitoes and some bird species without having any clear pathogenicity; outbreaks have been reported only in South Africa and in the Democratic Republic of the Congo (*5**,**6*). Before our study, no data relative to WNV circulation in horses in sub-Saharan Africa were documented, and WNV activity had never been reported in Chad or Gabon.

Highest (92%–97%) seroprevalence was found in the western and central parts of the Sahelian area (Dakar and N'Djamena). This area, characterized by a semiarid climate and vegetation of steppe and brush grass, is the most frequently involved area for WNV isolations in birds and mosquitoes (*7**,**13*). The seroprevalence was lower in the east of the Sahelian area (Djibouti, 9%), where the climate is arid and the vegetation is semidesert, and in the sub-Sahelian area (3%–30%), where the vegetation is tropical rain forest or woodland savanna in a humid or semihumid climate. That forest favors the sedentariness of birds has been documented (*14*). The migration of birds may certainly be enhanced in the Sahelian area; the introduction of WNV by migratory birds during their flight between Senegal and Europe has been suspected as a cause of the 1996 outbreak in Morocco (*10*). To estimate possibilities of incursions of WNV, especially in Eurasia, effects of environmental factors such as climate and vegetation on reservoir and vector populations in sub-Saharan Africa should be precisely studied.

## References

[1] Smithburn KC, Hughes TP, Burke AW, Paul JH. A neurotropic virus isolated from the blood of a native of Uganda. Am J Trop Med Hyg. 1940;20:471–92.

[2] Campbell GL, Marfin AA, Lanciotti RS, Gubler DJ. West Nile virus. Lancet Infect Dis. 2002;2:519–29. 10.1016/S1473-3099(02)00368-7 12206968

[3] Hubalek Z, Halouzka J. West Nile fever: a reemerging mosquito-borne viral disease in Europe. Emerg Infect Dis. 1999;5:643–50. 10.3201/eid0505.990506 10511520 PMC2627720

[4] Murgue B, Murri S, Zientara S, Durand B, Durand JP, Zeller HG. West Nile outbreak in horses in southern France, 2000: the return after 35 years later. Emerg Infect Dis. 2001;7:692–6. 10.3201/eid0704.010417 11585534 PMC2631744

[5] Zeller HG, Schuffenecker I. West Nile virus: an overview of its spread in Europe and the Mediterranean basin in contrast to its spread in the Americas. Eur J Clin Microbiol Infect Dis. 2004;23:147–56. 10.1007/s10096-003-1085-1 14986160

[6] Nur YA, Groen J, Heuvelmans H, Tuynman W, Copra C, Osterhaus AD. An outbreak of West Nile fever among migrants in Kisangani, Democratic Republic of Congo. Am J Trop Med Hyg. 1999;61:885–8. 10674664 10.4269/ajtmh.1999.61.885

[7] Centre Collaborateur OMS de Référence et de Recherche sur les Arbovirus. Flavivirus, West Nile: 282 souches identifiées. 1962 [updated 2005 Jul; cited 2006 Aug 17]. Available from http://www.pasteur.fr/recherche/banques/CRORA/virus/v010100.htm

[8] Murgue B, Murri S, Triki H, Deubel V, Zeller HG. West Nile in the Mediterranean basin: 1950–2000. Ann N Y Acad Sci. 2001;951:117–26. 10.1111/j.1749-6632.2001.tb02690.x 11797769

[9] Murgue B, Zeller H, Deubel V. The ecology and epidemiology of West Nile virus in Africa, Europe and Asia. Curr Top Microbiol Immunol. 2002;267:195–221. 10.1007/978-3-642-59403-8_10 12082990

[10] Schuffenecker I, Peyrefitte CN, El Harrak M, Murri S, Leblond A, Zeller HG. West Nile virus in Morocco, 2003. Emerg Infect Dis. 2005;11:306–9. 15752452 10.3201/eid1102.040817 PMC3320441

[11] De Madrid AT, Porterfield JS. A simple micro-culture method for the study of group B arboviruses. Bull World Health Organ. 1969;40:113–21. 4183812 PMC2554446

[12] Prangé A, Coussinier-Paris P, Davoust B, Cabre O, Gravier P, Sanson Y, Diagnostic différentiel de l'infection par le virus West Nile: intérêt de l'immunoblot. Proceedings of the 11th Actualités du Pharo Conference; 2004 Sep 9–11; Marseilles, France. Med Trop (Mars). 2004;64:293.

[13] Traore-Lamizana M, Zeller HG, Mondo M. Isolations of West Nile and Bagaza viruses from mosquitoes (Diptera: Culicidae) in center Senegal (Ferlo). J Med Entomol. 1994;31:934–8. 7815413 10.1093/jmedent/31.6.934

[14] Brown LH, Urban EK, Newman K, eds. The birds of Africa. Vol.1. 1st ed. New York: Academic Press; 1982.

